# Circulating Angiogenic Growth Factors in Diabetes Patients with Peripheral Arterial Disease and Exertional Leg Pain in Ghana

**DOI:** 10.1155/2017/2390174

**Published:** 2017-12-27

**Authors:** Kwame Yeboah, Jennifer A. Agyekum, Eric Kyei Baafour, Daniel A. Antwi, Afua B. Adjei, Vincent Boima, Ben Gyan

**Affiliations:** ^1^Department of Physiology, School of Biomedical & Allied Health Sciences, University of Ghana, Accra, Ghana; ^2^Department of Medical Laboratory Sciences, School of Biomedical & Allied Health Sciences, University of Ghana, Accra, Ghana; ^3^Department of Immunology, Noguchi Memorial Institute for Medical Research, University of Ghana, Accra, Ghana; ^4^Department of Chemical Pathology, School of Biomedical & Allied Health Sciences, University of Ghana, Accra, Ghana; ^5^Department of Medicine & Therapeutics, School of Medicine & Dentistry, University of Ghana, Accra, Ghana

## Abstract

**Objective:**

Peripheral arterial disease (PAD) is a common complication of diabetes, associated with impairment in angiogenesis. Angiogenesis is regulated by angiogenic growth factors such as angiopoietin 1 (Ang-1), Ang-2, and vascular endothelial growth factor (VEGF). We studied the association between angiogenic growth factors versus PAD and exertional leg symptoms in diabetes patients in Ghana.

**Method:**

In this cross-sectional study, ankle-brachial index was measured with oscillometrically and exertional leg symptoms were screened with Edinburgh claudication questionnaire in 140 diabetes patients and 110 nondiabetes individuals. Circulating levels of Ang-1, Ang-2, and VEGF were measured with immunosorbent assay.

**Results:**

The prevalence of PAD and exertional leg pain was 16.8% and 24.8%, respectively. Compared to non-PAD participants, PAD patients had higher VEGF levels [85.8 (37.5–154.5) versus 57.7 (16.6–161.1) *p* = 0.032] and lower Ang-1 levels [31.3 (24.8–42.6) versus 40.9 (28.2–62.1), *p* = 0.017]. In multivariable logistic regression, patients with exertional leg pain had increased the odds of plasma Ang-2 levels [OR (95% CI): 2.08 (1.08–6.41), *p* = 0.036].

**Conclusion:**

Diabetes patients with PAD and exertional leg pain have imbalance in angiogenic growth factors, indicating impaired angiogenesis. In patients with exertional leg pains, Ang-2 may be an important biomarker.

## 1. Introduction

Peripheral arterial disease (PAD) is the formation of atherosclerotic plaque in non-coronary vessel and preferentially affects the lower extremities. PAD is the third leading cause of atherosclerotic vascular death after coronary heart disease and stroke. Globally, it was estimated in 2013 that PAD affects more than 202 million individuals [1]. The prevalence of PAD in sub-Sahara African population is largely undetermined. Our previous studies indicated that PAD is common among non-smoking Ghanaians, especially in diabetes patients [2]. Arterial obstruction in PAD may be presented as leg pain during exertional activities, attributed to inadequate skeletal muscle perfusion to meet the increased metabolic demands [3]. The Edinburgh claudication questionnaire (ECQ) can be used to screen for symptoms associated with such symptomatic PAD [4]. However, more than 70% of PAD patients are asymptomatic and clinical signs may appear at late stages of the disease [5]. In such patients, ankle-brachial index (ABI), which is a simple, highly reproducible, noninvasive hemodynamic test, can be used to screen for PAD. ABI < 0.9 may also imply the presence of systemic atherosclerosis [6].

Angiogenic growth factors are endogenous molecules that regulate growth and development of the microvessels [7]. Most studies have reported vascular endothelial growth factor (VEGF), angiopoietin 1 (Ang-1), and angiopoietin 2 (Ang-2) to be potent regulators of microvascular remodelling in health and atherosclerotic diseases [8]. Ang-1 is expressed in non-endothelial cells, such as pericytes, smooth muscle cells, and fibroblast, whereas the major source of Ang-2 is from the endothelial cells [7]. In PAD patients, atherosclerotic occlusion results in ischemia of tissues distal to the plaque. This stimulates varying degrees of collateral blood vessel formation mediated by circulating angiogenic growth factors. Ang-2/VEGF promotes vessel sprouting and proliferation, whereas Ang-1 promotes circumferential growth required for vessel maturation and stabilization [8]. The degree of functional collateral blood vessel formation may alter the clinical manifestations of PAD. We, therefore, studied the levels of circulating angiogenic growth factors, Ang-1, Ang-2, and VEGF, in diabetes patients with PAD and leg pain on exertion. We hypothesize that, compared to individuals without PAD, PAD patients have imbalance in angiogenic growth factors.

## 2. Methods

### 2.1. Design and Setting

This study was conducted within the period of December 2012 to June 2013, at the Korle-Bu Teaching Hospital in Accra, which is a 1500-bed tertiary hospital and serves as the main referral hospital in Ghana. ABI was measured in all the study population, which were selected from two sources: (1) diabetes patients, selected systematically as every 3rd consecutive patient visiting the diabetes clinic and consented to take part in the study, and (2) nondiabetic individuals, invited from the surrounding communities and conveniently recruited into the study. All the study participants were categorised as PAD (ABI < 0.9) and non-PAD (ABI: 0.9–1.3) based on the ABI values. Individuals with stiff/incompressible arteries (ABI > 1.3), history/medication of CVDs, and those unable to comprehend and comply with the protocol requirements (psychological and/or cognitive disorders, failure to cooperate, and failure to sign the informed consent document) were excluded from the study. In all, 250 subjects, comprising 140 diabetes patients and 110 nondiabetic individuals, were screened for PAD. This study was conducted according to the tenets of the Declaration of Helsinki of 1975 (1983 revision) and was carried out with the approval of the University of Ghana Medical School Ethical and Protocol Review Committee (protocol ID number: MS-Et/M.2–P.4.10/2012-2013). All the study participants gave written informed consent after the procedures involved in the study were thoroughly explained to them.

### 2.2. Anthropometry and BP Measurement

Weight, height, waist, and hip circumferences were measured using standard protocol [9]. Briefly, body weight was measured twice using a homologated electronic scale (Seca 770) following due calibration (precision ± 0.1 kg), with the patient wearing light clothing with shoes removed. Height was also measured with a portable system (Seca 222) with the patient shoeless in the upright position. Body mass index (BMI) was calculated as weight (kg) divided by height squared (m^2^). Waist circumference was measured with nonelastic tape measure at the upper border of the iliac crest, parallel to the floor without compressing the skin. Blood pressure was measured three times, with a validated Blood Pressure Monitor (Omron 991X, Omron Health Care, Japan), at the right upper arm of participants with an appropriate cuff size, after at least 5 min rest, seated comfortably with arm and back support. Hypertension was defined as subjects with BP ≥ 140/90 mmHg and/or on antihypertensive medication.

### 2.3. ABI and Exertional Leg Pain Assessment

Ankle and brachial blood pressures were measured in all participants after a minimum of 5 min rest in a supine position on an examination table in a temperature controlled room using automated oscillometric method (Vasera 1500N, Fukuda-Denshi, Tokyo). The BP cuffs of the Vasera were applied to both arms and ankles to simultaneously measure BPs. ABI was calculated for each leg as the ratio systolic BP in the ankle divided by the higher of systolic blood pressure in the arm. ABI > 0.9 was considered normal and PAD was defined as ABI ≤ 0.9 in at least one leg.

Leg symptoms of patients were assessed using the ECQ and categorised based on previous work by Hirsch et al. [10]. The presence of exertional leg pain was defined as patients with classical intermittent claudication (exertional calf symptoms that do not begin at rest worsen when walking uphill or hurrying and resolve within 10 min of rest), atypical intermittent claudication (exertional calf symptoms that do not begin at rest but are otherwise not consistent with classical intermittent claudication), as well as rest pains (exertional leg symptoms that also begin at rest).

### 2.4. Biochemical Analysis

Blood samples were drawn in the morning, after 8–12 hours of overnight fasting. Fasting plasma glucose (FPG), 2-hour postglucose load plasma glucose (2 h PPG), total cholesterol, high-density lipoprotein cholesterol (HDL), triglyceride, and creatinine were analysed using BS 400 chemical autoanalyser (Mindray, China) and commercial reagents (Randox Laboratory Reagents, UK). Low-density lipoprotein (LDL) cholesterol levels were calculated using Friedewald's formula.

Serum levels of Ang-1, Ang-2, and VEGF were measured by sandwich enzyme-linked immunosorbent assay, using commercially available enzyme-linked immunosorbent assay kits (R&D Systems, Minneapolis, MN). The assays were performed according to the manufacturer's recommendations and the total interassay coefficient of variation for the three assays were <7 %. Lowest limit of the detection were 0.03 ng/ml for VEGF, 0.16 ng/ml for Ang-1, and 0.06 ng/ml for Ang-2.

### 2.5. Statistical Analysis

Continuous data were analysed with the Shapiro-Wilk test to determine their distribution. Variables with normal distribution were presented as mean ± standard deviation and analysed using Student's *t*-test. Variables with nonnormal distribution were presented as median and interquartile range and analysed using Mann–Whitney *U* test. Categorical data were analysed by the *χ*^2^ test. Multivariable logistic regression models were performed to compute adjusted and unadjusted odd ratios between (1) angiogenic growth factors versus PAD (low ABI) and (2) angiogenic growth factors versus leg pain on exertion.

## 3. Results

Diabetes patients had a higher proportion of hypertensives and had higher heart rate, FPG, total cholesterol, and LDL cholesterol levels, as well as lower HDL cholesterol levels. Compared to nondiabetes participants, diabetes patients had higher prevalence of PAD and low eGFR. For circulating angiogenic growth factors levels, compared to nondiabetes participants, diabetes patients had higher levels of plasma Ang-2 and VEGF and lower levels of Ang-1 (Table 1). The average duration of diabetes was 8.5 ± 6.9 years, higher in diabetes patients with PAD (12.1 ± 9.5 versus 6.4 ± 4.8 years, *p* = 0.028) or exertional leg pain (10.6 ± 7.1 versus 6.8 ± 5.3 years, *p* = 0.044), compared to patients without PAD or exertional leg pain, respectively. With respect to treatment of diabetes, 18 (12.9%) patients were on diet and lifestyle modification, 56 (40%) patients were on oral hypoglycaemic medication only and 66 (47.1%) were treated with both oral hypoglycaemics and injectable insulin. Compared to diabetes patients with PAD, patients without PAD (71.4% versus 10.9%) were more likely to be treated with oral hypoglycaemics and insulin [OR = 2.23 (1.08–4.59), *p* = 0.03].

**Figure 1:**
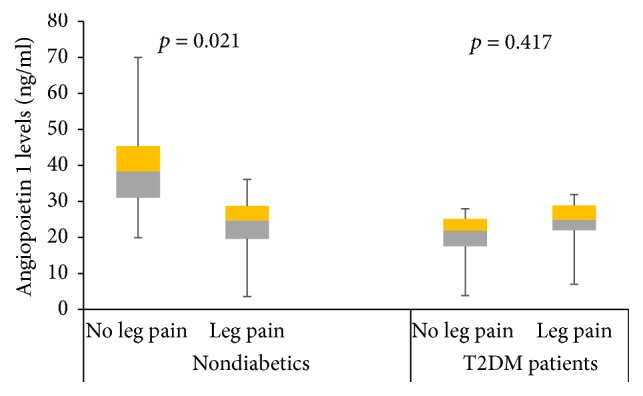
Circulating levels of angiopoietin 1 among patients with leg pains based on their diabetes status. Data presented as median interquartile range and analysed with Mann–Whitney test.

**Figure 2:**
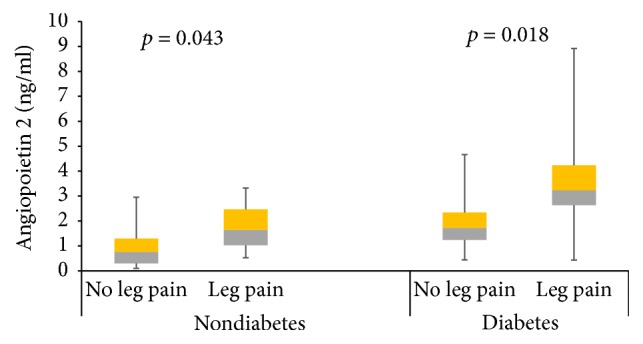
Circulating levels of angiopoietin 2 among patients with leg pains based on their diabetes status. Data presented as median interquartile range and analysed with Mann–Whitney test.

**Figure 3:**
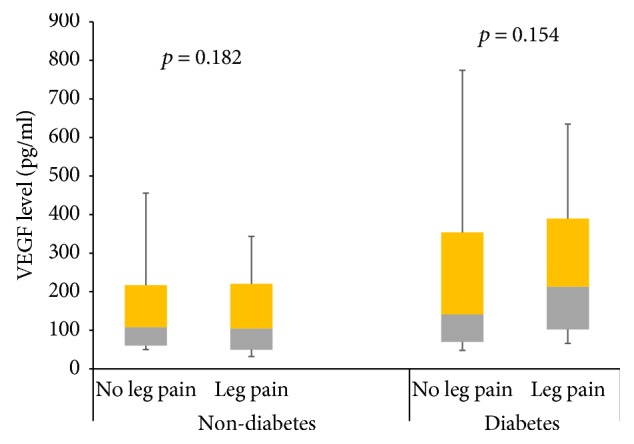
Circulating levels of VEGF among patients with leg pains based on their diabetes status. Data represented as median and interquartile range and analysed with Mann–Whitney test.

The overall prevalence of PAD (low ABI), in at least one leg, was 16.8%, with 14.4% of the participants having PAD in the right leg and 10.4% having PAD in the left leg. The overall prevalence of exertional leg pain was 24.8% in at least one leg, with 8.8% of the participants having both PAD and leg pain. Patients with PAD had higher pulse BP, higher proportion of leg pain, diabetes, and hypertension and are more likely to be former smokers and alcohol consumers. Compared to non-PAD participants, PAD patients had higher serum levels of VEGF and lower levels of Ang-1 (Table 2). Compared to participants without leg pain, nondiabetic participants with leg pain had lower levels of serum Ang-1, whereas Ang-1 levels were similar between diabetic participants with or without leg pain (Figure 1). In diabetes and nondiabetes participants, those with leg pain on exertion had higher levels Ang-2 (Figure 2). However, there was no difference in VEGF levels between diabetes and nondiabetes participants with or without leg pain (Figure 3).

In unadjusted logistic regression models, having PAD was significantly associated with odds of increase in VEGF and odds of decrease in Ang-1. However, this association was nonsignificant after adjusting the model for various covariates (Table 3). Patients with leg pain had an increase in odds of serum levels of Ang-1 and Ang-2 in unadjusted logistic regression model, but only Ang-2 remained significant after adjusting for various covariates (Table 4).

## 4. Discussion

The major findings of this study are that PAD patients had increased levels of VEGF and decreased levels of Ang-1; however, this was dependent on other CVD risk factors. Also, patients with leg pain on exertion have increased plasma levels of Ang-1 and Ang-2. The association between exertional leg pain and Ang-2 levels was independent of other CVD risk factors in regression model. Studies conducted in the American and British population have shown that patients with PAD have deranged levels of vascular growth factors. Similar to our findings, studies in the UK reported increased plasma VEGF levels in PAD patients [11, 12]. Contrary to the findings of Findley et al. [13] in PAD patients from the United States, we found plasma levels of Ang-2 was similar among PAD and non-PAD patients in our study.

Peripheral arterial disease is a major health care problem in diabetes patients in Ghana. We reported previously that the prevalence of PAD in diabetes patients in Ghana was 26.7%, using Doppler measured ABI [14]. In sub-Saharan Africa, ABI is not routinely performed to diagnose PAD, even in high-risk diabetes patients. PAD, therefore, is clinically underrecognized, and management is initiated when associated with symptomatic exertional leg pain. However, most patients with PAD are asymptomatic, and, hence, relying on symptoms before initiating treatment may be less advantageous [2, 14]. Moreover, the presence of PAD indicates generalised atherosclerosis in such patients. The use of ABI measurement as diagnostic test for PAD is based on the principle that atherosclerotic plaque within the peripheral arterial tree dampens blood flow and pressure towards tissues distal to the plaque, resulting in decreased tissue perfusion and tissue hypoxia. PAD would, therefore, affect capillary modelling and circulating levels of angiogenic growth factors [15, 16].

Similar to the findings of this study, imbalance in angiopoietins levels has been reported in diabetes patients elsewhere. Circulating Ang-2 and VEGF levels, but not Ang-1 levels, were reported to be associated with endothelial damage in diabetes patients, regardless of the presence of CVD. [17] In hyperlipidaemia patients in Japan [18], compared with nondiabetes patients, diabetes had increased Ang-2 levels, while Ang-1 levels were similar between the two patient groups. A population study in Asians indicates that increased levels of Ang-2 were associated with impaired glucose metabolism, diabetes, and hypertension [19]. Likewise, in the Anglo-Scandinavian Cardiac Outcome Studies, compared to nonhypertensives, hypertensive patients had higher levels of plasma Ang-1, Ang-2, and VEGF [20].

Capillary growth or regression is regulated by the balance between proangiogenic factors and antiangiogenic factors [8]. Ang-1 regulates vessel maturation and it mediates migration, adhesion, and survival of endothelial cells [21]. Ang-2 is known to cause disruption of connections in endothelial and perivascular cells and promotes cell death and vascular regression. However, in conjunction with VEGF, Ang-2 promotes development of new vessels [8, 21]. Imbalance in vascular growth factors in PAD patients affects angiogenesis in skeletal muscles. Contrary to studies conducted decades ago which reported similar capillarization between PAD patients and controls [22], recent studies have reported reduction in capillary density of muscles of the lower leg of PAD patients [23, 24]. Angiogenic factors, such as Ang-1 and VEGF, are required for functional neovascularization in adult tissue in PAD patients [25]. The findings of the current study indicate the imbalance in Ang-1 and VEGF levels in PAD patients, which may affect the ability of the leg muscular tissues to develop new capillaries to adapt to hypoxia induced by atherosclerotic plaque [26].

Compared to participants without leg pain, the levels of Ang-1 decreased, whereas Ang-2 levels increased, in participants with leg pain on exertion. The change was more pronounced in nondiabetes participants. Surprisingly, no change in VEGF level was observed. The reason for this observation may be a result of ethnic differences in vascular growth factors, which has been demonstrated in populations of Caucasians and African Caribbean origins [27]. Also, in our study, we assayed the serum levels of VEGF-A using pan-VEGF-A Elisa kits that cannot discriminate between various splicing variants of VEGF-A. Circulating levels of VEGF-A isoforms, arising from differential splicing of exon 8, have important consequence on angiogenesis; VEGF-A_XXXa_ isoforms are proangiogenic, while the VEGF-A_XXXb_ isoforms are antiangiogenic [15]. It has been reported that PAD patients have increased levels of antiangiogenic VEGF-A_165b_ splicing isoform, and corresponding reduced levels of VEGF-A_165a_ proangiogenic splicing isoform [16]. Leg pain on exertion reflects inadequate augmentation of skeletal muscle perfusion during exercise, an advanced form of PAD [13, 26]. Insufficient blood supply produced by arterial ischemia induces a complex program of vascular growth factors dysfunction, resulting in inadequate angiogenesis and collateral vessel formation [26]. Other studies have shown that imbalance in vascular growth factors can be corrected by pharmacological and nonpharmacological interventions [22, 24].

## 5. Limitations and Conclusion

The current guideline for PAD diagnosis recommends the use of continuous wave Doppler to measure ABI. In our study, however, ABI was measured using oscillometric method. The oscillometric method is convenient and operator-independent and measures all the limb pressures concurrently. Some studies have reported good concordance between Doppler and oscillometric methods of assessing ABI [28]. Others have reported that, compared to Doppler ABI, oscillometric ABI overestimates the prevalence of PAD. The use of oscillometric method of ABI assessment might be an important limitation in our study [29]. In addition, we did not measure the levels of various splicing variants of VEGF-A isoforms in our study, making the interpretation of circulating levels of VEGF-A difficult. Also, we cannot infer causality from the cross-sectional design of the study. Another important limitation is the inclusion of large numbers of diabetes patients in order to obtain enough PAD patients for analysis since diabetes affects angiogenic growth factors independent of PAD. All the same, this study has shown that PAD patients in sub-Saharan Africa have impaired vascular growth factors levels, an indication of impaired angiogenesis.

## Figures and Tables

**Table 1:** General characteristics of study participants by diabetes status.

	All participants (*n* = 250)	T2DM (*n* = 140)	Non-DM (*n* = 110)	*p*
Females, *n* (%)	125 (50)	63 (45)	62 (56.4)	0.15
Age, yrs	54.1 ± 10.2	53.7 ± 10.1	54.6 ± 10.3	0.54
Weight, kg	79.5 ± 14.9	79.9 ± 15.5	79 ± 14.3	0.672
Height, cm	166 ± 8.4	167 ± 8	164 ± 9	0.061
BMI, kg/m^2^	29.1 ± 5.7	28.9 ± 5.9	29.4 ± 5.5	0.571
Waist circumference, cm	98 ± 14	99 ± 12	96 ± 15	0.073
Waist-hip ratio	0.91 ± 0.11	0.92 ± 0.07	0.9 ± 0.14	0.382
Systolic BP, mmHg	139 ± 30	141 ± 26	135 ± 34	0.174
Diastolic BP, mmHg	83 ± 13	83 ± 13	82 ± 14	0.594
Pulse BP, mmHg	59 ± 14	59 ± 14	58 ± 13	0.485
Heart rate, bpm	71 ± 17	75 ± 13	65 ± 19	<0.01
FPG, mmol/l	6.9 ± 3.2	8.4 ± 2.9	5 ± 2.5	<0.01
2 h PPG, mmol/l	7.8 ± 1.4		7.8 ± 1.4	
Triglycerides, mmol/l	1.1 ± 0.5	1.1 ± 0.5	1.2 ± 0.6	0.586
Total cholesterol, mmol/l	4.7 ± 1.5	5.5 ± 1.4	3.9 ± 1.1	<0.001
HDL cholesterol, mmol/l	0.9 ± 0.2	0.7 ± 0.2	1.2 ± 0.4	0.025
LDL cholesterol, mmol/l	3.2 ± 1.4	3.9 ± 1.3	2.7 ± 1.4	<0.001
Serum creatinine mg/dl				
eGFR ml/min/1.73 m^2^	82.4 (73.1–104.9)	70.8 (51.4–82.1)	105.9 (76.2–118.7)	<0.001
Low eGFR, *n* (%)	43 (17.2)	34 (24.3)	9 (8.2)	0.001
Angiopoietin 1, ng/ml	38.2 (25.7–47.9)	36.1 (24.7–42.1)	41.1 (30–57.3)	0.01
Angiopoietin 2, pg/ml	740 (392–1107)	838 (473–1241)	597 (274–1005)	0.018
VEGF-A, pg/ml	63.2 (21.2–157.8)	72.2 (28–201.8)	48.4 (17.4–110.1)	0.025
VPT, mV	11.9 ± 6.2	15.1 ± 7.8	7.3 ± 3.8	<0.001
Right ABI	0.98 ± 0.18	0.92 ± 0.21	1.17 ± 0.13	0.135
Left ABI	0.97 ± 0.13	0.91 ± 0.12	1.15 ± 0.14	0.132
Right leg PAD	36 (14.4)	29 (20.7)	7 (6.4)	<0.001
Left leg PAD	26 (10.4)	21 (15)	6 (5.5)	<0.001
Leg pains on exertion	62 (24.8)	43 (37.7)	19 (17.3)	0.004

**Table 2:** Clinical characteristics of study participants by PAD status.

Characteristics	All participants (*n* = 250)	Non-PAD (*n* = 208)	PAD (*n* = 42)	*p*
Age, years	54.1 ± 10.2	53.6 ± 10.3	58 ± 8.8	0.062
Female, *n* (%)	127 (50.8)	98 (47.1)	17 (40.5)	0.142
Diabetes, *n* (%)	140 (56)	106 (51)	34 (80.9)	0.002
Hypertension, *n* (%)	152 (60.8)	116 (55.8)	36 (85.7)	<0.001
Leg pains	62 (24.8)	40 (19.2)	22 (52.4)	<0.001
Alcohol intake, *n* (%)	12 (4.8)	4 (1.9)	8 (19)	<0.001
Previous smokers, *n* (%)	47 (18.8)	22 (10.6)	25 (59.5)	<0.001
Body height, cm	166 ± 8.4	165 ± 8	166 ± 9	0.699
BMI, kg/m^2^	29.1 ± 5.7	29.1 ± 5.4	29.5 ± 7.5	0.735
Body fat, %	34.6 ± 12.6	35.1 ± 12.6	32.2 ± 12.9	0.318
Visceral fat, %	11.2 ± 4.3	11.2 ± 4	12.2 ± 6.2	0.301
Waist circumference, cm	98 ± 14	97 ± 13	101 ± 14	0.321
Waist-hip ratio	0.91 ± 0.11	0.91 ± 0.11	0.93 ± 0.08	0.287
Systolic BP, mm Hg	139 ± 30	139 ± 24	142 ± 55	0.629
Diastolic BP, mm Hg	83 ± 13	82 ± 13	89 ± 18	0.124
Pulse BP, mm Hg	59 ± 14	58 ± 13	68 ± 17	0.027
Mean BP, mm Hg	102 ± 16	102 ± 14	112 ± 21	0.06
Heart rate, bpm	71 ± 17	71 ± 15	71 ± 29	0.937
FPG, mmol/L	6.9 ± 3.2	6.8 ± 3.3	7.1 ± 2.7	0.673
2 h PPG, mmol/L	7.8 ± 4.1	7.6 ± 3.8	9.8 ± 7.1	0.221
Total cholesterol, mmol/L	4.7 ± 1.5	4.7 ± 1.5	4.7 ± 1.5	0.981
Triglycerides, mmol/L	1.1 ± 0.5	1.1 ± 0.5	1.1 ± 0.5	0.626
HDL, mmol/L	0.73 ± 0.22	0.73 ± 0.23	0.71 ± 0.15	0.695
LDL, mmol/L	3.3 ± 1.4	3.2 ± 1.5	3.4 ± 1.4	0.642
eGFR ml/min/1.73 m^2^	82.4 (73.1–104.9)	86.3 (72.2–106.1)	80.9 (71.4–101.8)	0.542
Low eGFR, *n* (%)	43 (17.2)	32 (15.4)	11 (26.2)	0.095
Vascular growth factors				
Ang-1, ng/ml	38.3 (25.7–47.9)	40.9 (28.2–62.1)	31.3 (24.8–42.6)	0.017
Ang-2, pg/ml	739.7 (391.7–1106.9)	732.2 (391.7–1098.8)	858.8 (416.7–1559.2)	0.174
VEGF-A, pg/ml	63 (21–158)	57.7 (16.6–161.1)	85.8 (37.5–154.5)	0.032
Ang-1/2	46.3 (27.6–90.6)	48.8 (29.7–93)	38.9 (20.9–69.7)	0.081

**Table 3:** Logistic regression models of peripheral arterial disease and vascular growth factors.

	Crude OR (95% CI)	*p*	Adjusted OR (95% CI)^*∗*^	*p*
Ang-1	0.91 (0.56–0.99)	0.024	0.84 (0.42–1.48)	0.096
Ang-2	1.56 (0.89–3.06)	0.532	1.92 (0.76–3.52)	0.427
VEGF	1.29 (1.07–1.97)	0.037	1.14 (0.92–2.13)	0.104
Ang-1/2	1.08 (0.66–1.84)	0.347	1.21 (0.51–2.41)	0.625

^*∗*^Adjusted for age, gender, alcohol status, previous cigarette smoking, BMI, waist-hip ratio, diabetes, hypertension, and total cholesterol. All the angiogenic growth factors were logarithmically transformed to improve skewness before analysis. Ang-1: angiopoietin 1; Ang-2: angiopoietin 2; VEGF: vascular-endothelial growth factor; Ang-1/2: angiopoietin 1/angiopoietin 2 ratio.

**Table 4:** Logistic regression models of leg pains and vascular growth factors.

	Crude OR (95% CI)	*p*	Adjusted OR (95% CI)^*∗*^	*p*
Ang-1	1.11 (1.02–2.26)	0.048	1.09 (0.59–2.01)	0.479
Ang-2	3.05 (1.14–8.94)	0.012	2.08 (1.08–6.41)	0.036
VEGF	1.48 (0.35–10.25)	0.747	0.69 (0.41–9.53)	0.78
Ang-1/2	1.16 (0.27–2.39)	0.239	0.8 (0.32–1.99)	0.631

^*∗*^Adjusted for age, gender, alcohol status, previous cigarette smoking, BMI, waist-hip ratio, diabetes, hypertension, and total cholesterol. All the angiogenic growth factors were logarithmically transformed to improve skewness before analysis. Ang-1: angiopoietin 1; Ang-2: angiopoietin 2; VEGF: vascular-endothelial growth factor; Ang-1/2: angiopoietin 1/angiopoietin 2 ratio.

## Abbreviations

PAD: Peripheral arterial disease
ABI: Ankle-brachial index
ECQ: Edinburgh claudication questionnaire
Ang: Angiopoietins
VEGF: Vascular endothelial growth factor.

## Additional Points

*Availability of Data and Materials.* The dataset supporting the conclusion of this study is available for systematic review and meta-analysis upon request.
